# Molecular crosstalk between cancer and neurodegenerative diseases

**DOI:** 10.1007/s00018-019-03428-3

**Published:** 2019-12-28

**Authors:** Jiyeon Seo, Mikyoung Park

**Affiliations:** 1 Center for Functional Connectomics, Brain Science Institute, Korea Institute of Science and Technology, Seoul, 02792 South Korea; 2 Department of Neuroscience, Korea University of Science and Technology, Daejeon, 34113 South Korea; 3 Center for Neuroscience, Brain Science Institute, Korea Institute of Science and Technology, Seoul, 02792 South Korea

**Keywords:** Age-related diseases, Cell death, Cell survival, Redox system, Glioma, Neurotoxicity

## Abstract

The progression of cancers and neurodegenerative disorders is largely defined by a set of molecular determinants that are either complementarily deregulated, or share remarkably overlapping functional pathways. A large number of such molecules have been demonstrated to be involved in the progression of both diseases. In this review, we particularly discuss our current knowledge on p53, cyclin D, cyclin E, cyclin F, Pin1 and protein phosphatase 2A, and their implications in the shared or distinct pathways that lead to cancers or neurodegenerative diseases. In addition, we focus on the inter-dependent regulation of brain cancers and neurodegeneration, mediated by intercellular communication between tumor and neuronal cells in the brain through the extracellular microenvironment. Finally, we shed light on the therapeutic perspectives for the treatment of both cancer and neurodegenerative disorders.

## Introduction

**Fig. 1:**
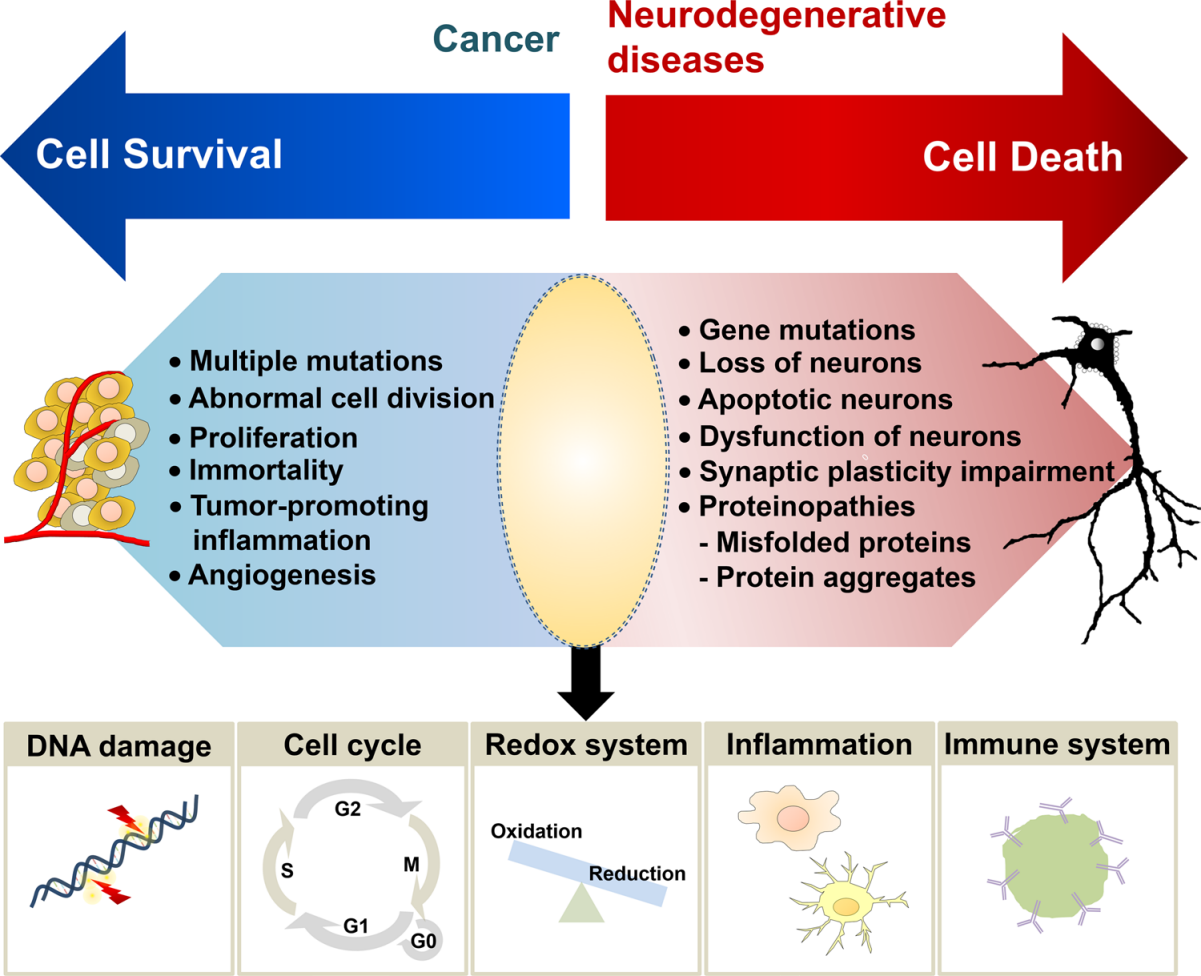
Featured hallmarks of cancer and neurodegenerative diseases. Pathophysiological features of two representative age-related diseases, cancer and neurodegenerative diseases are shown. Mechanisms are inversely undergone in these two diseases to lead to cell survival and cell death in cancer and neurodegenerative diseases, respectively. DNA damage, cell cycle aberrations, redox imbalance, inflammation, and immunity are closely associated as emerging shared characteristics between cancer and neurodegenerative diseases

Cancer and neurodegenerative diseases represent one of the most chronic physiological ailments. Aging, characterized by the deterioration of physiological functions necessary for survival and fertility, is considered as a major risk factor for the two disorders [1–3]. Cancer has been associated with generalized hallmarks such as sustenance of proliferative signaling, evasion of growth suppressors, resistance to cell death, acquisition of replicative immortality, induction of angiogenesis, and activation of invasion and metastasis. Interestingly, current research has indicated parameters such as deregulated cellular energetics and avoidance of immune destruction, as pertinent hallmarks. These features are effectuated by genome instability, mutations and/ or tumor-promoting inflammation [4–6] (Fig. 1). Neurodegeneration is characterized by dysfunction and loss of neurons [7], impairment of synaptic plasticity, proteinopathies, which include misfolded amyloid-β (Aβ) and tau in Alzheimer’s disease (AD), α-synuclein in Parkinson’s disease (PD), and their aggregates [8–11], as well as progressive muscle atrophy or muscle wasting, which causes memory deficits, cognitive failures, and movement disorders [7] (Fig. 1).

Inverse comorbidities between cancer and neurodegenerative diseases have been reported by many clinical and epidemiological studies [12–24]. In this light, molecular mechanisms that operate inversely in these two disorders (one leading to enhanced resistance to cell death and the other to a higher risk of cell death) would form effective means of diagnosis and prognosis at the physiological level (Fig. 1). A recent study, which performed transcriptomic meta-analyses of three neurodegenerative diseases (AD, PD, and schizophrenia) and three kinds of cancers (lung, prostate, and colorectal cancer) reported a significant overlap between the genes upregulated in the neurodegenerative diseases and downregulated in cancer, and between the genes downregulated in the neurodegenerative diseases and upregulated in cancer [16].

Multiple signaling pathways that regulate cell death and survival have been well investigated in tumorigenesis, including DNA damage, cell cycle aberrations, inflammation, immunity, and oxidative stress; these pathways have now been shown to be associated with neurodegenerative diseases as well [18, 25–31] (Fig. 1). In addition, aberrant expression or mutations in genes such as *α-synuclein*, *phosphatase and tensin homolog* (*PTEN*)*, PTEN induced kinase 1* (*PINK1; parkinsonism associated deglycase 6, PARK6*), *DJ-1* (*PARK7*), *leucine rich repeat kinase 2* (*LRRK2; PARK8*), *microtubule-associated protein tau* (*MAPT*), *amyloid precursor protein* (*APP*), *presenilin 1/2* (*PSEN1/2*), and *cyclin-dependent kinase 5* (*CDK5*), which play essential roles in neurodegeneration, are also observed in cancer [32].

**Fig. 2:**
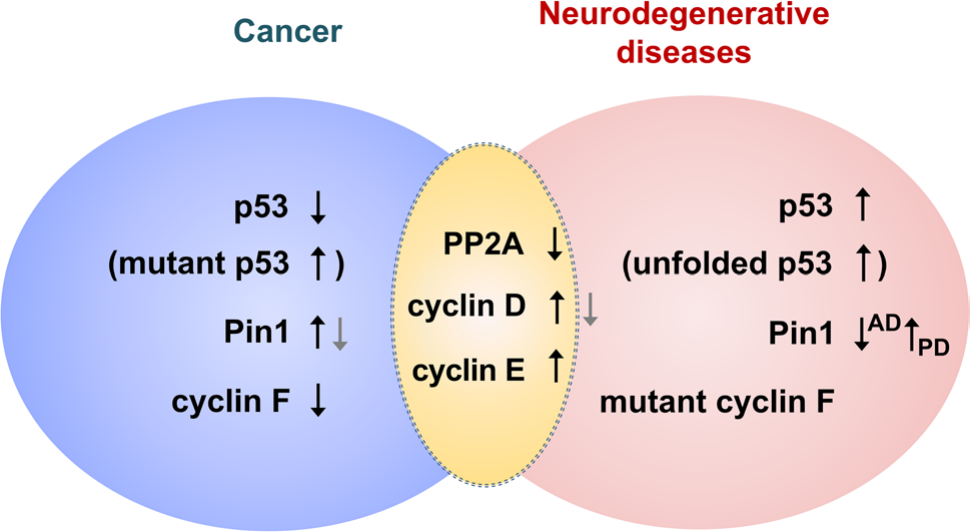
Changes in overlapping molecules in cancer and neurodegenerative diseases. Cyclin D and cyclin E are upregulated whereas protein phosphatase 2A (PP2A) is downregulated in both diseases. p53 is downregulated in cancer but inversely upregulated in neurodegenerative diseases. Peptidyl-prolyl *cis–trans* isomerase NIMA-interacting 1 (Pin1) is mainly upregulated in cancer and Parkinson’s disease (PD) but downregulated in Alzheimer’s disease (AD). Cyclin F is downregulated in cancer, while its mutant form is found in amyotrophic lateral sclerosis (ALS) and frontotemporal dementia (FTD). *NIMA* never in mitosis A

Over the past decade, accumulating evidences have demonstrated an intriguing relationship between cancer and neurodegenerative diseases. Better understanding of the relationship between the two will provide novel avenues for the study of these age-related diseases. In this review, we will discuss the current knowledge on the shared or distinct roles of overlapping molecules that have been significantly correlated with the pathophysiology of both cancer and neurodegenerative diseases, such as p53, cyclin D, cyclin E, cyclin F, peptidyl-prolyl *cis–trans* isomerase (PPIase) NIMA (Never in Mitosis A)-interacting 1 (Pin1), and protein phosphatase 2A (PP2A) (Fig. 2). In addition, we describe the inter-dependent regulation of brain cancers and neurodegeneration through intercellular communications between tumor and neuronal cells in the brain. Furthermore, this review provides some perspectives into the application towards pharmacological therapeutics for both cancer and neurodegenerative diseases.

## Overlapping molecules between cancer and neurodegenerative diseases

### p53

**Fig. 3:**
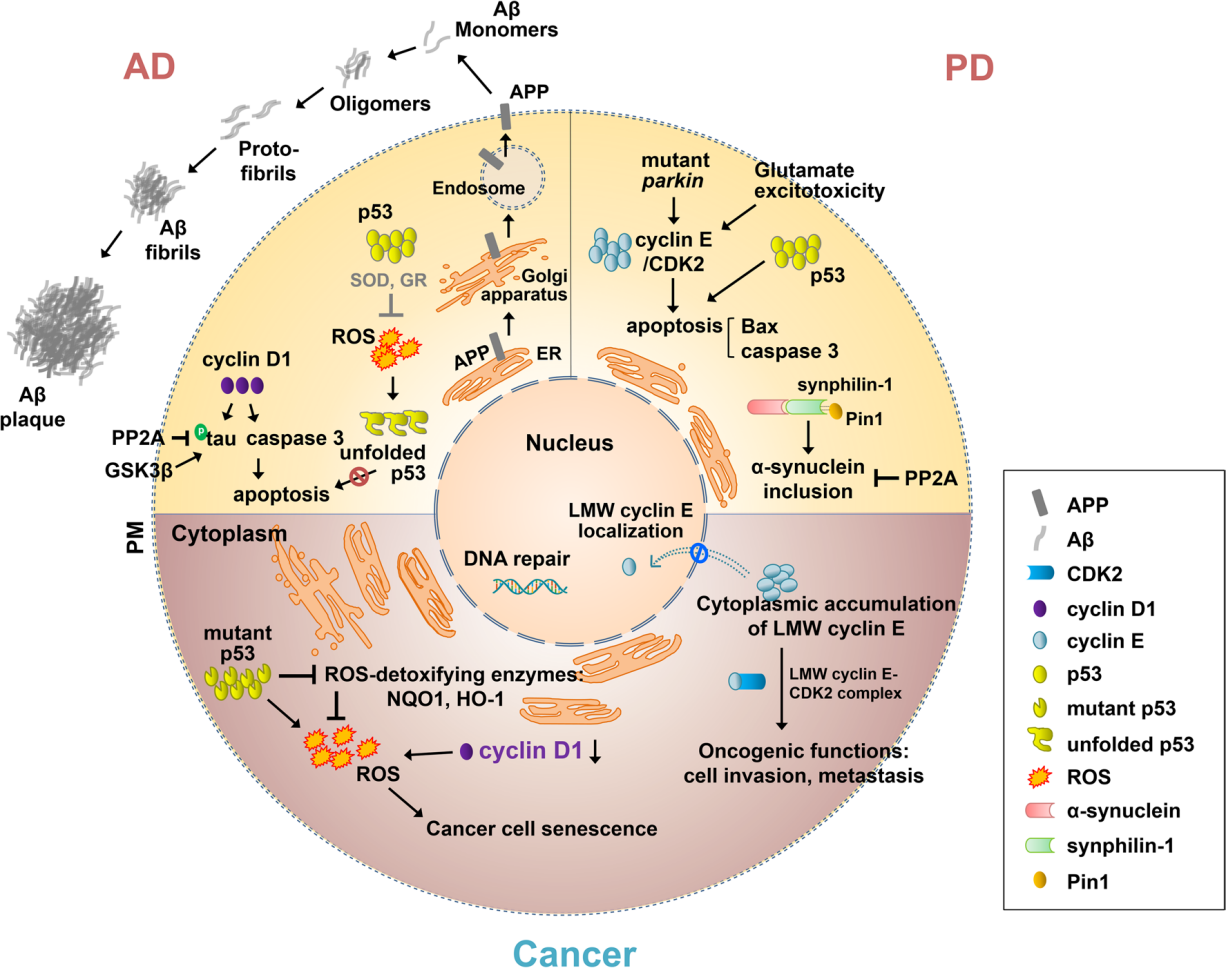
Mechanisms of overlapping molecules in cancer and neurodegenerative diseases. (Down) Mutant p53 inhibits the nuclear factor erythroid 2 (NF-E2)-related factor 2 (NRF2)-mediated antioxidant enzymes, and thus induces reactive oxygen species (ROS) production. Low-molecular-weight (LMW) cyclin E cannot translocate into the nucleus, and forms LMW cyclin E cyclin-dependent kinase 2 (CDK2) complex in the cytoplasm, thereby activating oncogenic functions, such as tumor cell invasion and metastasis. (Top left) Amyloid-β (Aβ), Aβ fibrils and plaques are formed from amyloid precursor protein (APP) via amyloidogenic processing. Cyclin D1 promotes tau phosphorylation and induces apoptosis through a caspase 3-mediated pathway. PP2A dephosphorylates phosphorylated tau, and glycogen synthase kinase 3 beta (GSK3β) phosphorylates tau. Reduced activity of superoxide dismutase (SOD) and glutathione reductase (GR) induces the increase of ROS production, which leads to the conformational change of p53, and this unfolded p53 is also observed in AD. (Top right) Mutant *parkin* and glutamate excitotoxicity increase cyclin E accumulation, which induces apoptosis. In addition, p53 also induces the upregulation of apoptotic proteins, such as Bcl-2 associated X (Bax) and caspases 3, which is observed in the PD brain. The interaction between Pin1 and synphilin-1 (an α-synuclein-binding protein) enhances the formation of α-synuclein inclusions, and this α-synuclein inclusion formation can be inhibited by PP2A. *ER* endoplasmic reticulum, *HO-1* heme oxygenase-1, *NQO1* quinone oxidoreductase 1, *PM* plasma membrane. Molecule name marked in purple indicates studies that involve exogenous proteins

The transcription factor p53 is an extensively studied tumor suppressor [33–35], and is known to be associated with around 50% of all human malignancies. In most of these cases, p53 gene has been reported to contain missense mutations [36–39]. The mutant p53 proteins no longer have tumor suppressor activity, and obtain several gain-of-functions such as invasion [40–48], enhanced migration [42, 49–52], anchorage-independent growth [53–58], propagation of cell cycle [59–65], cell survival and avoidance of cell death [66–76], genomic instability [77–82], and angiogenesis [83–85]. A commonly occurring mutant form of p53, p53-R273H, contributes to the impaired detoxification of reactive oxygen species (ROS) by decreasing the nuclear factor erythroid 2 (NF-E2)-related factor 2 (NRF2)-mediated expression of phase 2 ROS-detoxifying enzymes, quinone oxidoreductase 1 (NQO1) and heme oxygenase-1 (HO-1), which resulted in a reduced antioxidant response and imbalance of redox homeostasis in lung or colon cancer cells [70, 86, 87] (Fig. 3). Overexpression of another mutant, p53-G245D, upregulated a transcription factor called forkhead box protein M1 (FOXM1), which exerted oncogenic properties [88]. However, another study showed that the enhanced level of FOXM1 downregulates ROS levels by increasing antioxidant enzymes like superoxide dismutase (SOD) and thioredoxin-dependent peroxide reductase, peroxiredoxin 3 (PRDX3) [89]. These complicated results of mutant p53 on redox homeostasis could warrant more careful considerations when targeting dual factors p53 and redox regulation for the treatment of cancers. Furthermore, mutant p53 proteins are rather reluctant to degradation compared to wild-type p53 proteins, and thus the accumulated mutant p53 proteins are often a major therapeutic target for cancer treatment [36, 37, 85, 90–92].

Unlike the role of p53 in cancer, the level and activity of p53 in neurodegenerative diseases have been shown to be substantially increased [93–95]. Brains of AD patients and model mice showed increased levels of p53 [96–99] and apoptotic neuronal cell death [100–102]. In double transgenic AD mice that express the mutants of amyloid precursor protein/presenilin (APP/PS) and accumulate Aβ [103], cerebral gray matter displayed a positive correlation between the p53 level and accumulated Aβ level [104]. In addition, the triggering receptor expressed on myeloid cells 2 (TREM2), an AD-associated molecule, was reported to be upregulated positively in a p53-dependent manner in vitro [105]. Similar to AD, p53 level and activity were also increased in the brains of PD patients and model mice [106]. Specifically, the caudate nucleus, but not the substantia nigra, putamen, and cerebral cortex, of the PD patient brains showed a significantly enhanced p53 protein level [106]. The p53-dependent apoptosis-related proteins, such as Bcl-2 associated X (Bax) and caspase-3, were increased in the PD brains [107, 108].

Genetic mutations in p53 have not been reported for neurodegenerative diseases. However, functionally compromised variants of p53 such as those with an altered tertiary structure (called unfolded p53 or conformational mutant p53) have been distinctly observed in AD patients [109, 110] and in older APPswe/PS1-A246E AD transgenic mice [111], but not in non-AD individuals, including PD patients [109]. In human neuroblastoma cell line SH-SY5Y overexpressing APP, an increased level of unfolded p53 was observed. This was shown to be associated with a lack of p53 pro-apoptotic activity (Fig. 3) and an impairment in neuronal responses against acute cytotoxic injury [112].

Oxidative imbalance has also been demonstrated as a distinctive feature in neurodegenerative diseases [113–119]. This imbalance has been observed to be mediated by reduced activities of SOD and glutathione reductase during AD [113]. Importantly, it was noted that the reduced enzyme activity of SOD corresponded with increased levels of unfolded p53, suggesting that ROS possibly contributes to p53 conformational change in AD [113] (Fig. 3). In a PD model induced by the treatment of 1-methyl-4-phenylpyridinium (MPP +), the expression of sestrin-2, an antioxidant protein, was increased by MPP + -induced p53 activation, and such enhanced expression of sestrin-2 protects cells against ROS, suggesting a novel role of p53 in PD [119]. Therefore, counteracting oxidative stress or improving cellular antioxidative properties would provide effective therapeutic alternatives for neurodegenerative disorders.

### Cyclins

Many studies have demonstrated that dysregulated destruction of cell cycle regulators, many of which play a role in either tumor suppression or tumorigenesis, is tightly linked to cancer initiation and progression. Cyclins are known to regulate cell cycle by modulating the activity of cyclin-dependent kinases (CDKs). Deregulation of the cell cycle through changes in the activity of cell cycle CDKs or their regulators form essential determinants of human cancers [120–127].

Beyond their role in cell cycle regulation, cyclins contribute immensely to the cellular aspects of the terminally differentiated neurons [128]. Cell cycle-independent roles of cyclins, including cyclin E and cyclin Y, have been reported in postmitotic neuronal physiology [129–131]. Cyclin E deficiency has been shown to reduce the number of synapses and spine volume, and also to impair long-term potentiation and memory [131]. Knockdown of cyclin Y in hippocampal neurons has been reported to enhance activity-dependent synaptic delivery of α-amino-3-hydroxy-5-methyl-4-isoxazolepropionate (AMPA) receptors and long-term potentiation [129]. Interestingly, such aberrations in the cell cycle components and the resulting neuronal cell death have also been found in neurodegeneration and neurodegenerative diseases [132–135].

#### Cyclin D

**Fig. 4:**
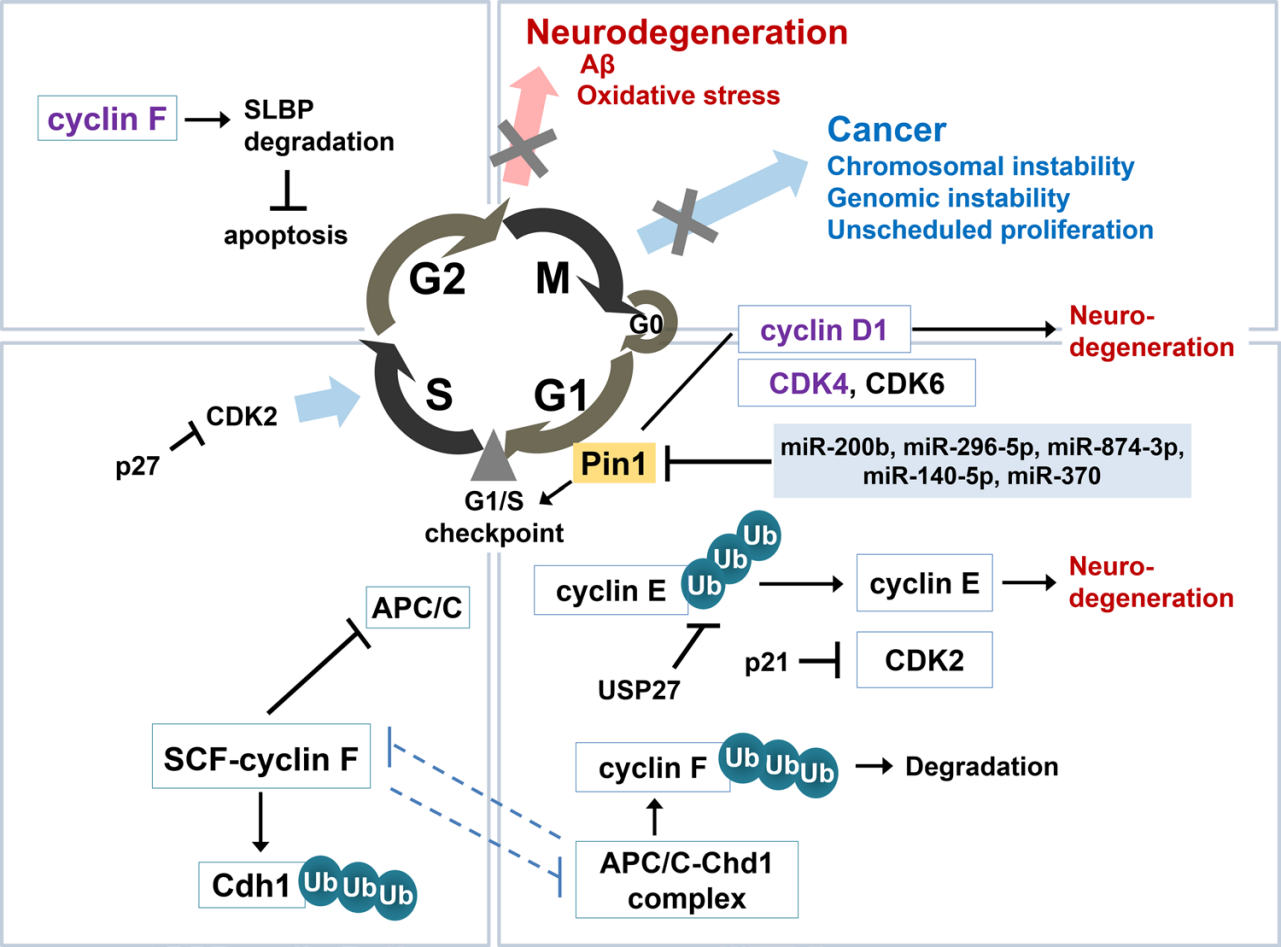
Cell cycle and cyclins, such as cyclin D, cyclin E, and cyclin F in cancer and neurodegenerative diseases. Dysregulated cell cycle is tightly related with cancer initiation as well as neurodegeneration. Cyclin D1, associated with CDK4 and CDK6, modulates the entry from quiescence (G_0_) to the G1 phase of the cell cycle, and is also linked to the progression of neurodegeneration. Cyclin E plays a role in the initiation of DNA replication at the G1/S checkpoint, and also causes neurodegeneration in the postmitotic neurons. Ubiquitin-specific peptidase 27 (USP27), a cyclin E interactor, increases the stability of cyclin E by inhibiting the ubiquitination and subsequent degradation of cyclin E. SCF-cyclin F and APC/C-Cdh1 are controlled by a negative reciprocal feedback circuit, which controls S phase entry. In addition, cyclin F interacts with stem-loop binding protein (SLBP) and promotes SLBP degradation during G2. Blockade of the interaction between cyclin F and SLBP increases apoptosis upon genotoxic stress in G2 phase. Pin1 is a positive regulator of cyclin D1, and both Pin1 and cyclin D1 are upregulated in many cancers. Pin1 expression is negatively regulated by small non-coding microRNAs (miRNAs), including miR-200b, miR-296-5p, miR-874-3p, miR-140-5p, and miR-370. *SCF* Skp1–Cul1–F-box, *APC/C* anaphase promoting complex/cyclosome, *Chd1* Cdc20 homologue 1. Molecule names marked in purple indicate studies that involve exogenous proteins

Cyclin D, which controls the entry from the quiescence (G_0_) to G_1_ phase of the cell cycle [136], and its associated CDKs, CDK4 and CDK6, are overexpressed and hyperactive, respectively, in most tumors [124, 137] (Fig. 4). Mutant cyclin D1 knockin mice, in which CDK4 and CDK6 are not activated, were resistant to breast tumors initiated by the activated *erbB-2* oncogene [122, 127]. Mice lacking cyclin D1 were also resistant to breast cancers induced by the *erbB-2* and *ras* oncogenes, but were sensitive to breast cancers induced by other oncogenes like *c-myc* or *Wnt-1*. Furthermore, knockdown of cyclin D1 induced oxidative imbalance by elevating intracellular ROS levels in cancer cells, which promoted the senescence of cancer cells through a retinoblastoma-independent pro-senescence pathway [138]. These investigations suggest that anti-breast cancer therapy targeting cyclin D1 could be very specific to breast cancers depending on the activated pathways [127].

CDK4, a cyclin D-associated CDK, was observed to be increased in the brains of AD patients [139]. In addition, the upregulation of cyclin D was reported to be linked to increases of phosphorylated tau and caspase 3, which led to apoptosis in cultured hippocampal neurons, suggesting that cyclin D increment could be a crucial factor in the progression of neurodegenerative pathology [140] (Fig. 3). Conversely, in *cyclin D1-*deficient mutant mice, phenotypically characterized by small eyes with thin retinas, a reduced proliferation of retina cells and an increased photoreceptor cell death were observed [141]. In addition, stimuli inducing cortical neuronal degeneration reduced the protein level of cyclin D1 [142]. Recently, neuronal gain- or loss-of-function of cyclin D/CDK4 in *Drosophila* caused neurodegeneration [143]. In addition, cyclin D/CDK4-mediated neurodegeneration was shown to be mediated by altered mitochondrial function and an accompanying increase in ROS [143].

#### Cyclin E

Cyclin E plays a role in the initiation of DNA replication at the G1/S checkpoint [144] and is a regulatory subunit of CDK2 [145]. Cyclin E was reported to be overexpressed in many types of cancers, such as breast cancer [146], non-small-cell lung cancer [147], colorectal carcinomas [148], lymphomas [149], acute myelogenous leukemia [150], gastric carcinomas [151], and osteosarcoma [152]. Over 10% of female transgenic mice overexpressing cyclin E developed mammary carcinoma at around 8–13 months of age [153]. Tumorigenesis mediated by cyclin E overexpression likely involves genomic instability [154–157] as cyclin D1 overexpression also induces genomic instability [158]. Ubiquitin-specific peptidase 27 (USP27) was recently identified as a cyclin E interactor, and was reported to increase cyclin E stability through the negative regulation of cyclin E ubiquitination [159] (Fig. 4). Depletion of USP27 inhibited the migration and metastasis of hepatocellular carcinoma and the tumor growth, suggesting that USP27 is a novel therapeutic target for cancers involving cyclin E [159].

Besides the 50 kDa full-length cyclin E, tumor-specific low-molecular-weight (LMW) cyclin E isoforms, ranging from 33 to 45 kDa, have been found to be accumulated in cancer cells [146, 160–166]. LMW cyclin E has mostly lost its N-terminal nuclear localization signal [167], which results in the cytoplasmic accumulation of cyclin E [168] (Fig. 3). It exerts oncogenic functions through properties such as hyperactivation of CDK2 due to more stable LMW cyclin E/CDK2 complex formation [169], the resistance of LMW cyclin E/CDK2 complex to inhibitors p21 and p27 [170], altered substrate interactions [171], and cytoplasmic novel interactions that differ from the full-length cyclin E [172]. Like cytoplasmic LMW cyclin E, excessive cytoplasmic cyclin D1 also exerts oncogenic functions by promoting tumor cell invasion and metastasis [173–175] through cytoplasmic interactions [175, 176].

Cyclin E is a substrate of the parkin E3 ubiquitin ligase. Mutations in the *parkin* gene are the most common cause of PD, and upregulates cyclin E and CDK2 [177–179]. Glutamate excitotoxicity has also been implicated in PD [180, 181]. Like *parkin* mutations, the glutamatergic excitotoxin kainate treatment increased cyclin E accumulation in cultured neurons, and this increase was further enhanced in parkin knockdown neurons, which resulted in the promotion of apoptosis (Fig. 3). Conversely, parkin overexpression retarded the cyclin E accumulation in cultured neurons treated with excitotoxin, and protected the neurons from the kainate-induced excitotoxicity [182]. Cyclin E expression is also related to AD [183–185], for example, expression of cyclin E in the brain induced cell cycle activation and led to neurodegeneration of postmitotic neurons in a *Drosophila* tauopathy AD model [184] (Fig. 4). In addition, Aβ treatment in cultured neurons increased ROS production and activated the mammalian target of rapamycin (mTOR) complex 1, thereby leading to the expression of cell cycle regulatory proteins such as cyclin D1/CDK4 and cyclin E/CDK2 [185].

#### Cyclin F

Cyclin F, also known as F-box only 1 (FBXO1), was first reported as the F-box family of proteins, which contain an F-box motif [186]. F-box proteins are the substrate-recognition subunits of Skp1–Cul1–F-box (SCF) E3 ubiquitin ligase complexes, and thus in the SCF-cyclin F complex, cyclin F recognizes target proteins and mediates the ubiquitination of target proteins for proteolysis [187, 188]. Cyclin F is also a member of the cyclin family; however, unlike other cyclins, which regulate the cell cycle in concert with the activity of their associated CDKs, cyclin F does not require CDK activity to regulate cell cycle-associated functions. The SCF-cyclin F complex controls the cell cycle through a tightly regulated ubiquitin-mediated proteolysis of centrosomal protein of 110 kDa (CP110), which prevents centrosomal duplications [189]. In addition, cyclin F in S phase regulates the ubiquitination and subsequent degradation of Cdc20 homologue 1 (Chd1), a substrate adaptor protein of the anaphase promoting complex/cyclosome (APC/C), while cyclin F in G1 phase is ubiquitinated and subsequently degraded by the APC/C-Chd1 complex [190] (Fig. 4). In addition, cyclin F interacts with stem-loop binding protein (SLBP) through Arg97 (R97) and Leu99 (L99) in SLBP, and regulates SLBP degradation during G2 [191]. Disruption of the interaction between SLBP and cyclin F by expressing SLBP (RL97/99AA) in G2 led to increased apoptosis upon genotoxic stress [191]. The crucial roles of F-box proteins, including cyclin F, in tumorigenesis are gradually becoming acknowledged owing to their pivotal roles in such cases of cell cycle regulation and genome stability [189, 192]. Recently, it was discovered that cyclin F is upregulated under metabolic stress conditions and inhibits tumorigenesis mediated by an oncogenic mutant form of isocitrate dehydrogenase 1 (IDH1), IDH1-R132H, in glioma [193]. Indeed, it was also reported that cyclin F is downregulated in hepatocellular carcinomas, and low cyclin F expression is correlated with poor survival and recurrence-free survival of hepatocellular carcinoma patients [194], supporting that, unlike other cyclins, cyclin F acts as a tumor suppressor and could be further investigated as a promising prognostic marker for hepatocellular carcinoma.

A recent study using whole-exome sequencing identified mutations in the cyclin F gene, *ccnf*, in the relatives of patients with amyotrophic lateral sclerosis (ALS) and frontotemporal dementia (FTD) [195], which have a common pathological feature of aberrant accumulation of ubiquitinated transactive response (TAR) DNA-binding protein 43 (TDP-43) [196–198]. These findings drive the need to investigate whether TDP-43 is a substrate of the SCF-cyclin F E3 ubiquitin ligase complex. An ALS/FTD-causing pathogenic mutation in cyclin F at amino acid position 621 from serine to glycine (Cyclin F-S621G) was shown to increase the specific ubiquitination at lysine-48 of proteins, which led to the accumulation of lysine-48-ubiquitinated proteins and the impairment of autophagic degradation [199], indicating autophagy to be a degradative mechanism underlying the pathogenesis of ALS/FTD. The roles of cyclin F, which acts as a cyclin as well as an F-box protein, have not been explored in this context, and thus can be further investigated to understand their relevance in mediating neurodegenerative diseases.

### Pin1

Pin1 is a regulatory protein of cyclin D1, which is a major regulator of G1 checkpoint progression. Like cyclin D1, Pin1 has been reported to be overexpressed in various cancers, including breast, colon, liver, and lung cancers. Further, cyclin D1 and Pin1 expression levels have been shown to correlate positively in such cancers [200, 201], thereby indicating Pin1 as a potential tumor-promoting factor. Accordingly, Pin1 expression and tumor progression have also been positively correlated in brain, breast, cervical, colon, liver, and prostate cancers [201–204].

Pin1 is a transcriptional target of the E2 factor (E2F). The E2F promotes Pin1 expression by binding to the E2F-binding sites of the Pin1 gene promoter [205]. In addition, several studies have demonstrated that small non-coding microRNAs (miRNAs), including miR-200b in breast tumor [206], miR-296-5p in prostate cancer [207], miR-874-3p [208] and miR-140-5p [209] in hepatocellular carcinoma, and miR-370 in esophageal squamous cell carcinoma [210], negatively regulate Pin1 expression (Fig. 4). In other words, suppression of such Pin1-targeting miRNAs leads to Pin1 overexpression in various cancers.

Alongside the tumor-promoting function, Pin1 has also been suggested to bear conditional tumor suppressor activity. Pin1 binds to and negatively regulates the protein expression levels of cyclin E [211, 212], whose overexpression mediates tumorigenesis and involves genomic instability [154–157] (Fig. 4). Many therapeutic studies for treating cancers have developed Pin1 inhibitors based on the fact that Pin1 is a generally recognized tumor-promoting factor [213–226]. Considering that Pin1 has also been reported to have a tumor suppressing function, Pin1-directed inhibitors must be carefully implicated in cancers.

In another paradigm, Pin1 has been known to bind to phosphorylated tau in normal and AD brain extracts, and soluble Pin1 protein has been found to be negligible in AD brains [227]. Pin1 facilitates the dephosphorylation of tau by PP2A [228]. Accordingly, Pin1 expression is inversely correlated with neurofibrillary hyperphosphorylated tau aggregates in AD [229]. Furthermore, *Pin1* knockout mice caused tau hyperphosphorylation and tau filament formation [229], and also enhanced amyloidogenic APP processing and selectively increased insoluble Aβ in brains [230]. Similarly in the synaptopathy aspect, Pin1 proteins are decreased in the synapses of AD patients and AD mice brains, and blocking Pin1 activity causes the degradation of a major postsynaptic density organizer, Shank3, resulting in the disruption of synapse structure and thus plasticity. These data indicate that loss of Pin1 activity could lead to deficits in synapse function and plasticity during AD development [231]. Taken together, Pin1 plays a pivotal role in protecting neurodegeneration, and thus could be used as a promising therapeutic target for AD.

Unlike in AD, Pin1 is upregulated in a 1-methyl-4-phenyl-1,2,3,6-tetrahydropyridine (MPTP)-induced PD mouse model and human PD brains [232]. Pin1 was reported to be involved in Lewy body formations in PD [233]; it locates to 50–60% of the Lewy bodies, which are cytosolic inclusions containing α-synuclein aggregates, in PD patient brains [233]. In addition, Pin1 interacts with an α-synuclein-binding protein synphilin-1 [234], which resultantly enhances the interaction between α-synuclein and synphilin-1 and thus the formation of α-synuclein inclusions [233] (Fig. 3). Given that Pin1 is downregulated and upregulated in AD and PD, respectively, precise modulations of Pin1 levels depending on the biological contexts might be one of the crucial factors to be considered in differential therapeutic strategies for treating AD and PD.

### PP2A

PP2A, a member of serine/threonine protein phosphatase, is a tumor suppressor [235–238] and a master regulator of the cell cycle known to dephosphorylate over 300 substrates related to the cell cycle [239]. Partial reduction of PP2A-Aα subunit expression to ~ 50% of normal levels induced anchorage-independent growth and tumorigenicity, whereas over 63% reduction of PP2A-Aα expression resulted in apoptosis [236]. In addition, a tenfold reduction of PP2A-Aα expression level was observed in almost half of the glioma samples studied [240]. A cancer-associated mutation in the PP2A-Aα subunit, PP2A Aα-E64D, increased the incidence of lung cancer by 50–60% in mice [241], further supportive of PP2A as a tumor suppressor (Fig. 2). Another mutation of PP2A-Aα, PP2A-Aα-W257G, was shown to promote cancer cell migration [242]. Apart from the PP2A-Aα subunit, mutations in the PP2A-B55α regulatory subunit have been identified in prostate cancer [243].

The phosphorylation level of proteins maintained by the activity of kinases and phosphatases is an important factor for regulating brain function, and PP2A is the most important phosphatase in the brain [244, 245]. One of the main hallmarks of AD is tau hyperphosphorylation [244, 246], which has 3–4-fold higher levels of tau phosphorylation compared to control brains [247]. Consistent with this notion, the reduced activity and expression of ABαC subunit of PP2A, the major tau phosphatase [248–250], which consists of a scaffolding A subunit, a regulatory B subunit, and a catalytic C subunit [251], were observed in AD brains, but not in non-AD dementias [252, 253]. In contrast, enhanced activation of glycogen synthase kinase 3 beta (GSK3β), a major tau kinase [248, 250, 254], was found to increase tau phosphorylation [250] (Fig. 3). Decreased activity and expression of PP2A-C subunit in AD were not only reported to be involved in tau hyperphosphorylation, but also suggested to be responsible for the activation of c-jun N-terminal kinase (JNK), which could lead to Aβ overproduction [255, 256]. Accordingly, two endogenous inhibitors of PP2A, I1(PP2A) and I2(PP2A), were upregulated in the neocortex by in situ hybridization in AD brains, and were suggested to be involved in the hyperphosphorylation of tau in AD [257].

The PP2A-B55α regulatory subunit serves as a major phosphatase for α-synuclein and prevents its accumulation; thereby restricting the key element of PD pathology [258, 259]. In addition, it has been demonstrated that α-synuclein regulates PP2A activity [260–263], and low activity of PP2A was reported in PD [259, 264] (Fig. 3). Taken together, balanced phosphorylation and dephosphorylation of proteins are critical for physiology, and in particular, reverting PP2A activity ultimately to dephosphorylate tau or α-synuclein could be a promising therapeutic strategy for AD or PD treatment [265, 266].

The shared mechanisms in both cancer and neurodegeneration involve activating kinases and inactivating protein phosphatases. For instance, in brain tumors, PP2A-Aα subunit levels have been found to be reduced in 8 out of 23 glioblastomas, 10 out of 19 oligodendrogliomas, and 7 out of 16 anaplastic oligodendrogliomas [240]. Further, PP2A-Aα subunit mutations were found to contribute to cancer development and tumorigenicity [236]. The most frequent PP2A-Aα mutation, R183W, has been shown to lack the ability to suppress tumor growth, and lead to decreased sensitivity of tumors towards MEK inhibitors [267]. Alongside, PPP2R2C, which encodes a gamma isoform of the subunit B55 subfamily, was also reported to be downregulated in various glioma cell lines and glioma patients [268]. Overexpression of PPP2R2C suppressed cancer cell proliferation by inhibiting the activity of S6K in the mTOR pathway, and further promoting the binding of PP2A-C with S6K, indicating PP2A as a potent tumor suppressor in human brain cancer [268]. Further investigation on the detailed mechanisms underlying PP2A downregulation in gliomas, which leads to neurodegeneration would provide better information for PP2A-based drug development for cancer and neurodegenerative diseases.

## Brain tumors and neurodegeneration: Intercellular communications between cancer and neuronal cells

As discussed in the previous section, molecules such as p53, cyclins, Pin1 and PP2A play important roles in the deregulation of homeostatic pathways in respective cell types, leading to cancers and neurodegeneration. Besides the intracellular actions of such molecules, recent studies have shed light on another viewpoint that cancer and neurodegeneration can affect each other by the communication between tumor cells and neuronal cells in the brain.

**Fig. 5:**
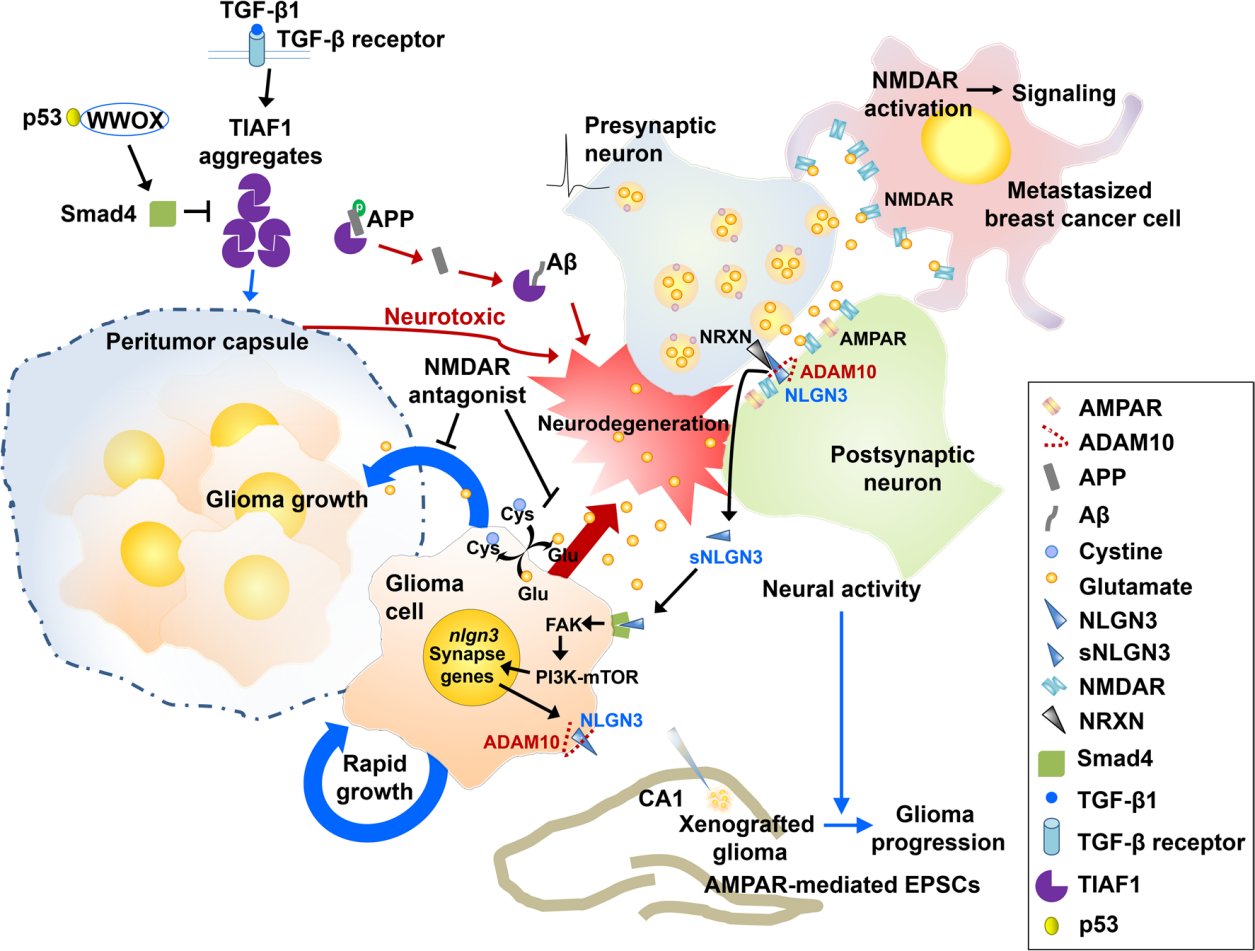
Reciprocal regulation between brain cancer and neuronal cells in the brain. Excessive glutamate (Glu) secreted from glioma cells leads to neurodegeneration as well as glioma progression. NMDA receptors (NMDARs) on the metastasized breast cancer cells receive glutamate, and promote breast-to-brain cancer metastasis. Neuroligin-3 (NLGN-3) is cleaved by ADAM10 and released from a postsynaptic neuron. This secreted soluble neuroligin-3 (sNLGN-3) acts as a mitogen for glioma cells, thereby fostering glioma progression through a FAK and PI3K–mTOR signaling pathway. Transforming growth factor β (TGF-β)1-induced anti-apoptotic factor (TIAF1) aggregates, which can form a peritumor capsule, cause neurotoxicity while suppressing tumor progression through the interaction with Smad4, WW domain-containing oxidoreductase (WWOX) and p53. Neurotoxicity can also be caused by the formation of TIAF1-Aβ complex. *ADAM10* a disintegrin and metalloproteinase 10, *AMPAR* AMPA receptor, *APP* amyloid precursor protein, *Cys* cystine, *FAK* focal adhesion kinase, *mTOR* mammalian target of rapamycin, *NRXN* neurexin, *Smad4* mothers against decapentaplegic homolog 4

Several studies have demonstrated that malignant primary brain tumor glioma cells secrete excessive glutamate via the cystine/glutamate antiporter xCT (SLC7A11) [269], which generates a toxic microenvironment for the neurons lying in the vicinity of the glioma, thereby inducing excitotoxicity, neuronal cell death, and neurodegeneration [270–273] (Fig. 5). In addition, gliomas implanted into the striata of adult rats have shown high glutamate release, rapid growth of the glioma, and neuronal degeneration in the vicinity of the tumor [272]. This effect was found to be reduced by blocking the glutamate receptor, *N*-methyl-d-aspartate (NMDA) receptor, with its antagonist MK801 or memantine [272], indicating that glutamate-releasing glioma cells mediate neurodegeneration by generating excessive glutamate excitotoxicity in the vicinity of the glioma. With this knowledge, potential therapeutic effects of antagonizing tumor-secreted glutamate or its receptors can be considered. These findings are further supportive of a positive correlation between brain tumors and AD [274, 275].

Besides the cancer cell-driven regulation of neurodegeneration, studies suggesting the neuronal regulation of brain cancer have also been demonstrated [276–279]. A recent study reported that presynapse-releasing glutamate is implicated in invasive tumor growth in breast cancer metastasized to the brain [279]. Breast cancer metastasized to the brain express NMDA receptors that may be activated by glutamate released from the presynapse, and aid in promoting the breast-to-brain cancer metastasis [279]. The paracrine action of glutamate is achieved by forming pseudo-tripartite synapses, composed of a cancer cell, a presynapse, and a postsynapse [279] (Fig. 5). In a patient-derived pediatric glioblastoma xenograft model, optogenetically induced neuronal activity promoted the proliferation and growth of the glioma in vivo [276, 278]. This glioma growth was found to be mediated by activity-dependent cleavage and secretion of the synaptic adhesion molecule neuroligin-3 from a postsynaptic neuron or oligodendrocyte precursor cell. This cleavage is carried out by a disintegrin and metalloproteinase, ADAM10 [278]. The secreted soluble neuroligin-3 can act as a mitogen for the glioma, inducing focal adhesion kinase (FAK), phosphoinositide 3-kinase (PI3K)-mTOR pathway, expression of neuroligin-3 and other synapse genes, leading to the proliferation of the glioma cells [276–278] (Fig. 5). Interestingly, patient-derived glioma xenografted into the CA1 region of hippocampal circuit was found to exhibit AMPA receptor-mediated excitatory postsynaptic currents (EPSCs) on the glioma, and form structural synapses with neurons [277]. The glioma progression was promoted through the integration of electrical and synaptic features of the glioma into neural circuits in the brain [277].

The microenvironment of brain cancer is also governed by transforming growth factor β (TGF-β)1-induced anti-apoptotic factor (TIAF1), found to be aggregated at the interface between metastatic cancer cells, such as metastatic small-cell lung cancer cells and metastatic lung adenocarcinoma, forming a protective peritumor capsule, that can be toxic to neurons [280] (Fig. 5). TIAF1 aggregates have been found in the hippocampi of both non-demented humans and AD patients, along with Aβ and tumor suppressors, such as Smad4 and WW domain-containing oxidoreductases (WWOX or WOX1) [280, 281]. TIAF1 aggregation suppresses anchorage-independent growth, metastasis, and tumor progression, while inducing apoptosis and cell death, which may lead to neurodegeneration [280, 281]. Consistently, a TIAF1/WWOX/p53 triad was found to suppress cancer progression [280, 282], but caused brain protein aggregation in the brain due to functional antagonism of p53 to WWOX-mediated cancer suppression, which lead to neurodegeneration [282]. Unlike TIAF aggregates, zinc finger-like protein that regulates apoptosis (Zfra) and bind tau and Aβ in the AD hippocampus, was reported to suppress melanoma-mediated neurodegeneration in the hippocampus and cortex [283]. More detailed investigation on the intracellular, extracellular or intercellular mechanisms of where and how TIAF1 and Zfra exert their actions in establishing communication between brain cancer cells and neuronal cells would be interesting.

## Discussion and perspectives

Many epidemiological studies have demonstrated an inverse correlation between the two age-related diseases, cancer and neurodegenerative diseases [22–24], and this intriguing correlation was restricted to certain types of cancers and neurodegenerative diseases. Indeed, in the case of schizophrenia, varying degrees of risk for different types of cancers have been reported [284, 285]. For instance, patients with schizophrenia have shown an increased, marginal, and decreased risk in colon, breast, and respiratory cancer, respectively [285]. Many studies, as described in this review, have revealed the shared roles of overlapping molecules involved in both cancers and neurodegenerative diseases. However, the underlying mechanisms for the two are very distinct, wherein cancers escape cell death while neurodegeneration occurs towards cell death (Fig. 1). Therefore, it would be conceivable that individuals afflicted with a neurodegenerative disease may have a reduced chance of developing certain types of cancers and vice versa. Given the molecular overlap of both diseases, studies in the fields of cancer and neurodegeneration would provide mutual benefits for each other. Because both diseases are closely associated with genetic mutations, it would be valuable to investigate the correlations of the genetic mutations which are found in one disease and also affect the other disease. For this, large amounts of intensive epidemiological studies investigating the incidence of one disease in the population that is affected by the other disease would need to be performed.

Besides clinical and epidemiological studies, which indicate an inverse association between cancer and neurodegenerative diseases [14, 18, 24, 31, 286, 287], studies on shared molecular mechanisms between cancer and neurodegenerative diseases are increasing [16, 288]. Further in-depth investigations into the cellular and molecular mechanisms related to distinct or shared features targeting the molecular crosstalk between cancer and neurodegeneration will assist in the development of additional biomarkers and new therapeutics. Because of the inverse associations between the two diseases with shared molecules in their pathological processes, the therapeutic development in cancer research may lead to the identification of prognostic markers even for both cancer and neurodegeneration, which could potentially result in improved treatments for both disorders. Indeed, over the last decade, drug repositioning from anticancer agents to medicine for neurodegenerative diseases or in the opposite direction has been applied to develop novel therapeutics to overcome these two aging-related diseases with success or failure [289, 290].

Cyclin D and cyclin E are upregulated in both cancer and neurodegenerative diseases, while PP2A is downregulated in both diseases. In addition, cyclin F is downregulated in cancer, and functionally mutated cyclin F is found in neurodegenerative diseases. p53 is downregulated in cancer but upregulated in neurodegenerative diseases, while Pin1 is upregulated in cancer and PD but downregulated in AD (Fig. 2). Overall, it seems that such overlapping molecules between cancer and neurodegenerative diseases may play important roles in pathophysiology and physiological functions differentially in various contexts, depending on the stage or severity of disease and molecular characteristics.

Several studies have demonstrated that inhibition of Pin1 effectively suppresses the growth of various cancer cells [291, 292], and is considered as a promising target for cancer treatment. Indeed, many small molecule inhibitors targeting Pin1 have been developed [215, 293, 294], that exhibit anticancer activities [225, 295]. All-trans retinoic acid (ATRA), a target drug used for acute promyelocytic leukemia (APL), binds to the substrate binding site of Pin1 and thus inhibits Pin1 activity in breast cancer [225]. Juglone, a compound produced by walnut trees, covalently modifies the catalytic core of Pin1 [215, 293], and inhibits multiple cancer cells [291, 296]. API-1, a small molecule targeting the PPIase domain of Pin1, suppresses the proliferation and migration of hepatocellular carcinoma cells [292]. KPT-6566 covalently binds to the PPIase catalytic core of Pin1, and selectively inhibits and degrades Pin1 [213]. Such Pin1 inhibitors that reduce Pin activity would not be directly applicable to treat AD because Pin1 deficiency contributes to AD, and Pin1 expression is inversely correlated with tauopathy and AD [230]. However, a compensatory activation or upregulation of Pin1 has been found in mild cognitive impairment, critically indicating that Pin1-based therapeutics needs to be considered depending on the course of AD [297].

In addition, intracellular organelles that regulate the balance between cell survival and death, are also governed by Pin1 in cancer and apoptotic neurons [298–300]. Activated p53, under genotoxic stress, regulates apoptosis-related Bax and Puma expression [301]. Pin1 binds to the activated p53 in the cytoplasm, which promotes the translocation of Pin1 to the mitochondrial membrane, where Pin1 binds to the Bcl-2 homology 3 (BH3)-only protein, Bcl-2-interacting mediator of cell death (Bim)-extralong (BimEL), and mediates neural-specific mitochondrial pro-apoptotic activity [299, 300]. As discussed, Pin1 can be either pro- or anti-apoptotic depending on the cellular context, and therefore, the role of Pin1 in mitochondria-driven apoptosis could provide a direct mechanical link between cancer and neurodegeneration. Therefore, future research in the field should prioritize the investigation of the sophisticated cellular and molecular mechanistic details between cancer and neurodegenerative diseases. Such work will provide a detailed checklist for the development and repositioning of therapeutics, and by unraveling the inverse association between cancer and neurodegenerative diseases, ultimately contribute to personalized medicine and treatment.

## Abbreviations

Aβ: Amyloid-β
AD: Alzheimer’s disease
ALS: Amyotrophic lateral sclerosis
AMPA: α-Amino-3-hydroxy-5-methyl-4-isoxazolepropionate
APC/C: Anaphase promoting complex/cyclosome
APP: Amyloid precursor protein
ATRA: All-trans retinoic acid
APL: Acute promyelocytic leukemia
Bax: Bcl-2 associated X
BH3: Bcl-2 homology 3
Bim: Bcl-2-interacting mediator of cell death
BimEL: Bim-extra long
CDK: Cyclin-dependent kinase
Chd1: Cdc20 homologue 1
CP110: Centrosomal protein of 110 kDa
E2F: E2 factor
EPSCs: Excitatory postsynaptic currents
ER: Endoplasmic reticulum
ERK: Extracellular signal-regulated kinase
FAK: Focal adhesion kinase
FBXO1: F-box only 1
FOXM1: Forkhead box protein M1
FTD: Frontotemporal dementia
GR: Glutathione reductase
GSK3β: Glycogen synthase kinase 3 beta
HD: Huntington’s disease
HO-1: Heme oxygenase-1
IDH1: Isocitrate dehydrogenase 1
JNK: C-jun N-terminal kinase
LMW: Low-molecular-weight
LRRK2: Leucine rich repeat kinase 2
MAPT: Microtubule-associated protein tau
miRNA: MicroRNA
MPP +: 1-Methyl-4-phenylpyridinium
MPTP: 1-Methyl-4-phenyl-1,2,3,6-tetrahydropyridine
mTOR: Mammalian target of rapamycin
NRF2: Nuclear factor erythroid 2 (NF-E2)-related factor 2
NIMA: Never in mitosis A
NMDA: *N*-methyl-d-aspartate
NQO1: Quinone oxidoreductase 1
PARK6: Parkinsonism associated deglycase 6
PD: Parkinson’s disease
Pin1: Peptidyl-prolyl *cis**–**trans* isomerase NIMA-interacting 1
PI3K: Phosphoinositide 3-kinase
PINK1: PTEN induced kinase 1
PM: Plasma membrane
PPIase: Peptidyl-prolyl *cis**–**trans* isomerase
PP2A: Protein phosphatase 2A
PRDX3: Peroxiredoxin 3
PSEN1/2: Presenilin 1/2
PTEN: Phosphatase and tensin homolog
ROS: Reactive oxygen species
SCF: Skp1–Cul1–F-box
SLBP: Stem-loop binding protein
Smad4: Mothers against decapentaplegic homolog 4
SOD: Superoxide dismutase
TAR: Transactive response
TDP-43: TAR DNA-binding protein 43
TGF-β: Transforming growth factor β
TIAF1: TGF-β1-induced anti-apoptotic factor
TREM2: Triggering receptor expressed on myeloid cells 2
USP27: Ubiquitin-specific peptidase 27
WWOX or WOX1: WW domain-containing oxidoreductase
Zfra: Zinc finger-like protein that regulates apoptosis
